# Vaccinating Sons against HPV: Results from a U.S. National Survey of Parents

**DOI:** 10.1371/journal.pone.0115154

**Published:** 2014-12-26

**Authors:** Jaime L. Taylor, Greg D. Zimet, Kelly L. Donahue, Andreia B. Alexander, Marcia L. Shew, Nathan W. Stupiansky

**Affiliations:** Section of Adolescent Medicine, Department of Pediatrics, Indiana University School of Medicine, Indianapolis, Indiana, United States of America; The Ohio State University, United States of America

## Abstract

**Purpose:**

The quadrivalent HPV vaccination was approved for use in males ages 9 to 26 in 2009 and recommended for routine administration in 2011. The purpose of this study was to uncover predictable commonalities amongst parents who chose to vaccinate their 11–17 year old sons against HPV.

**Methods:**

We compiled data from a U.S. national sample of 779 parents with sons 11–17 years old using a web-based survey to gather information about behavioral and sociodemographic factors which predicted receipt of 1 or more HPV vaccine doses based on parental report. Predictors were first modeled individually for univariable associations. Significant predictors (p<0.10) were combined in a multivariable model.

**Results:**

In the adjusted model, independent predictors included receipt of flu vaccination, health insurance coverage and sexual health topic discussions with sons. Sons who had received a flu shot in the last two years more frequently received at least one dose of the vaccine (OR 1.82; 95% CI 1.45–2.26). Sons covered by private health insurance had decreased odds of HPV vaccination (OR 0.56 95% CI 0.37–0.83). Lastly, parents who had discussed sexual health topics with their sons were more likely to vaccinate (OR 1.61; 95% CI 1.37–1.89).

**Conclusions:**

Male vaccination rates in the U.S. have increased, but males continue to be under-immunized. Utilization of health care is an important factor in HPV vaccine uptake; therefore, health care providers should use every contact as an opportunity to vaccinate. Communication about sexual health topics may provide a forum for parents and health care providers to have conversations about HPV vaccination as those more comfortable discussing these topics may also be more comfortable discussing HPV vaccination.

## Introduction

Human papillomavirus (HPV) is the most common sexually transmitted infection. [1] There are known causal associations between HPV and genital and anal cancers as well as genital warts and cancers of the head and neck. [2] The quadrivalent HPV vaccination protects against the most common HPV strains that cause HPV-related diseases: 6, 11, 16, and 18. It was approved for use in females in 2006 and, in 2009, it was approved for use in males ages 9 to 26. The recommendation for routine administration in males came in 2011. [3] Despite these recommendations, vaccination rates remain low. The most recent U.S National Immunization Survey-Teen (NIS-TEEN) data on adolescent vaccination indicates that 53.8% of females and 20.8% of males received at least one dose of HPV vaccine. [4] The NIS-TEEN collects data by list-assisted random-digit-dialing telephone survey followed by a mailed survey to children’s immunization providers [5].

The approval for use and consistent recommendations for HPV vaccination in males followed those for females, therefore, most research to date has concentrated on females. This research has focused largely on predictors of acceptability (i.e. attitudes surrounding vaccination and/or intention to vaccinate) and uptake (i.e. the actual act of getting the vaccine) [6] whereas most existing research regarding vaccination among males has focused solely on vaccine acceptability, largely because the recommendation to routinely vaccinate males is relatively recent.

Research published on females has suggested that parents who have previously vaccinated their adolescent daughters against HPV have more favorable attitudes toward HPV vaccine use in males. [7] Consistent with female vaccination data [8], [9], parents who consider religion more important are less willing to vaccinate their sons. [7] Parents who were more comfortable discussing new vaccines with their sons, and those with more liberal political views, were also more accepting of the HPV vaccine. [10] Additionally, it has been shown that males who have been vaccinated with other adolescent-timed vaccines, specifically the meningococcal vaccine, are more likely to have initiated the HPV vaccine [11], which is consistent with previous literature on female vaccination [12].

In one of the few studies published on HPV vaccine uptake in males, males whose mothers have received the influenza vaccine and pap smears are more likely to have been vaccinated. [13] Reiter et al showed that fathers are less likely than mothers to vaccinate and that health care provider (HCP) recommendation predicted vaccine uptake in males, similar to findings in adolescent females. [14] While vaccine uptake in males continues to increase, the percentage of males vaccinated remains low. There is currently insufficient data on predictors of male vaccine initiation. A recently published paper based on this dataset explored parents’ reported reasons for non-vaccination of sons. [15] The purpose of the current study was to examine parental beliefs, communication and vaccine-related health care utilization factors as potential predictors of HPV vaccination initiation of 11–17 year old sons. The importance of this study lies in the ability to discover why parents choose to vaccinate their sons.

## Methods

### Participants

We collected web-based survey data from a U.S. national sample of parents and legal guardians with sons 11–17 years of age (targeted age of the HPV vaccine). Using a survey research company (Survey Sampling International or SSI), data were drawn from U.S. adults in July and August of 2012. SSI maintains a national panel of adults in the U.S. and facilitates access to survey respondents for market and behavioral research. Invitations are sent at random to panel members fitting the study protocols. Those who volunteer are then entered into a lottery to win a monetary prize. Each panel member may participate in a maximum of four surveys annually. The respondents’ identities remain anonymous to study designers. Though the participants in this study were recruited nationally, they are not a nationally-representative sample.

For this study, emails were first sent to potential participants meeting inclusion criteria which consisted of having at least one son in the household between the ages of 11 and 17. Only one parent per household was asked to respond regarding only one son. Initially, 1322 panelists responded. 802 parents met eligibility criteria and agreed to participate in the study. There were 779 surveys completed with 758 of these respondents indicating the HPV vaccination status of their sons. If there was more than one son meeting inclusion criteria, parents were prompted to answer questions thinking of the youngest son in the designated age range. The nature of this study allowed for waiving of written informed consent which, along with study protocols, was approved by the Institutional Review Board at Indiana University-Purdue University, Indianapolis.

### Measures

The survey used for this study was designed to gather information pertaining to parental demographics (race, age, gender, education, employment, etc.), religious service attendance, political views, son’s health insurance and health care utilization as well as recent influenza vaccination history and parental discussion of sexual health topics with their sons. These specific variables were chosen for their relationship with acceptance and initiation of HPV vaccination in prior research. [8], [11], [12], [16]–[19] For HPV vaccination status, parents were asked to indicate how many HPV shots their son had received, with responses choices of “none”, “one”, “two”, or “three”. For analysis a bivariate outcome variable was created: no doses vaccine versus one or more doses of vaccine.

Parents reported the frequency of religious service attendance to measure how involved they were in their particular religious organizations based on a 5-point response scale of “rarely or never” to “more than once a week.” Parents were asked to describe their political views as “conservative,” “liberal,” or “middle of the road” which have been shown previously to be associated with vaccine acceptability. [8] Son’s health insurance type was chosen by parents as either private or non-private, which included Medicaid, other state funded, self-pay, other and unknown. Parents reported on sons’ recent healthcare utilization (i.e., whether or not their son was taken to a doctor, nurse, or other health care provider in the last year) and the number of flu shots their son received in the last 2 years. Using a 4-point response format (“not at all” to “a great deal”), parents also reported on the amount of time they had spent in the last 6 months discussing sexual health topics, such as using condoms to prevent STDs, with their sons.

### Analysis

Correlations were examined among all potential independent variables to assess for collinerarity. Logistic regression models were used to examine the outcome of receipt of 1 or more doses of HPV vaccine. Univariable models examined each of the behavioral and sociodemographic predictors with HPV vaccination outcome. Predictors with significant univariable associations (defined as p<0.10) were then included in a multivariable, adjusted model. All analyses were performed using SPSS 21. [20] Please refer to the supplemental material for data pertinent to this model (S1 Manuscript).

## Results

Both male and female parents completed the survey and were similarly represented at 48.5% and 51.5%, respectively. The mean age of parents completing the survey was 42.2 years (Standard Deviation = 11.5). Most parents were non-Hispanic White (73.8%), followed by non-Hispanic Black (10%), Hispanic (9.8%), and other race/ethnicity (6.5%). Sons had a mean age of 13.8 years (Standard Deviation = 2.0). The majority of sons (88.5%) had seen an HCP in the last year, and 63.8% had received at least one flu shot in the last two years. See Table 1 for a complete description of the sample. Of the 758 parents who provided information about their son’s HPV vaccination status, 162 (21.4%) indicated that one or more doses of the vaccine had been administered, 73 (9.7%) indicated that two or more doses of the vaccine had been administered and 24 (3.2%) indicated that 3 doses had been administered.

**Table 1:** Sample characteristics reported by parents (N = 779)*.

		Received 1 or MoreDoses (N = 162)	Received NoDoses (N = 596)
Variable	% or Mean (SD)	% or Mean (SD)	% or Mean (SD)
**Parent gender (N = 721)**			
Male	48.5%	47.6%	48.8%
Female	51.5%	52.4%	51.2%
**Parent age in years (N = 779)**	42.2 (11.5)	40.0 (12.0)	42.8 (11.3)
**Parent Ethnicity (N = 779)**			
White	73.8%	63.0%	77.2%
African American	10.0%	14.8%	8.7%
Hispanic	9.8%	16.0%	7.6%
Asian	3.6%	3.1%	3.9%
Other	2.9%	3.1%	2.6%
**Frequency of attendance at religious services (N = 763)**			
Rarely or never	38.4%	35.8%	39.3%
Few times a year	18.9%	24.7%	17.1%
1–3 times a month	14.2%	13.6%	14.3%
Once a week	21.1%	16.7%	22.3%
More than once a week	7.5%	9.3%	6.9%
**Political views (N = 756)**			
Conservative	29.0%	22.0%	30.8%
Middle of the road	52.1%	57.2%	51.3%
Liberal	18.9%	20.8%	17.9%
**Son’s age in years (N = 779)**	13.77 (1.95)	13.78 (1.88)	13.75 (1.97)
**Son visited health care provider in past year (N = 763)**			
Yes	88.5%	93.8%	87.2%
No	11.4%	6.2%	12.8%
**Number of flu shots son received in past 2 years (N = 759)**			
0	36.2%	16.5%	41.5%
1	22.4%	27.2%	20.8%
2	41.4%	56.3%	37.6%
**Son’s health insurance (N = 721)**			
Private	61.3%	50%	65%
Non-Private	38.7%	50%	35%
**Frequency of discussion with son about sexual health,** **such as the use of condoms to prevent STDs (N = 761)**			
Not at all	29.3%	15.4%	33.2%
Somewhat	21.9%	16.7%	23.3%
A moderate amount	25.0%	30.2%	23.5%
A great deal	23.8%	37.7%	20.0%
**Son’s HPV vaccination status (N = 758)**			
No doses or don’t know	78.6%	–	–
1 or more doses	21.4%	–	–

*Differences in N represent missing cases.

Table 2 shows the correlations among all predictors. As parent’s age increased, their political views became more conservative (r = −0.12, p<0.05), their religious service attendance increased (r = 0.09, p<0.05), and their sons were more likely to have private insurance (r = 0.07, p<0.05). The more liberal parental political views, the more likely they were to speak to their son regarding sexual health (r = 0.07, p<0.05). The more conservative parental political views, the more likely sons were to have private insurance (r = −0.10, p<0.05). As son’s age increased there was an attendant increase in sexual health discussions with the parent (r = 0.29, p<0.05) but a decrease in HCP visits (r = −0.11, p<0.05). These correlations, though statistically significant, all reflected small effect sizes, suggesting no issues of multicollinearity in multivariable analyses.

**Table 2:** Correlation between variables.

Variable	Parentgender	Parentage	Ethnicminority	Religiousserviceattendance	Politicalviews	Son’sage	Visitedhealth careprovider	Numberof flushots	Son’shealthinsurance	Sexual healthdiscussion
Parent gender	1.00	–0.04	–0.03	–0.04	0.08*	0.04	0.09*	–0.05	–0.10*	–0.02
Parent age		1.00	–0.24*	0.09*	–0.12*	0.21*	0.06	0.00	0.07*	0.02
Ethnic minority (1 = yes; 0 = no)			1.00	0.06	0.15*	–0.03	–0.07†	0.06	–0.15*	0.01
Frequency of religious service attendance				1.00	–0.31*	0.01	0.05	0.04	0.05	0.03
Political views					1.00	0.02	0.00	0.05	–0.10*	0.07*
Son’s age						1.00	–0.11*	–0.06	–0.00	0.29*
Son visited health care provider in past year							1.00	0.12*	0.01	0.07†
Number of flu shots received in past 2 years								1.00	–0.03	0.10*
Son’s health insurance									1.00	–0.03
Frequency of sexual health discussion										1.00

*p<0.05.

† *p*<0.10.

Univariable logistic regression analyses indicated that vaccine initiation was modestly, but inversely related to parental age (OR 0.98; 95% CI 0.96–0.99). Black (OR 2.08; 95% CI 1.23–3.53) or Hispanic (OR 2.61; 95% CI 1.54–4.42) parents were more likely than White parents to initiate vaccination. Sons who were evaluated by health care providers sometime in the last year more frequently received at least one dose of the vaccine (OR 2.22; 95% CI 1.12–4.40) as did those who had received a flu shot in the last two years (OR 1.82; 95% CI 1.45–2.26). Son’s with private health insurance were less likely to have initiated vaccination than those without private insurance (OR 0.55; 95% CI 0.38–0.79). There was an association between more reported discussion of sexual health topics and greater likelihood of first dose uptake (OR 1.61; 95% CI 1.37–1.89). Parent gender, religiosity (measured by religious service attendance), and sons’ age were not significant predictors of HPV vaccine initiation. Multivariable logistic regression was performed to include all variables in the model which were significant at p<0.10 in the univariable models. With the exception of parental age and political views as well as son’s visit to an HCP, all significant bivariate predictors remained independent predictors of HPV vaccine initiation in the adjusted, multivariable model (see Table 3).

**Table 3:** Factors associated with sons’ receipt of 1 or more doses of HPV vaccine.

Variable	Univariate OR (95% CI)	Multivariate OR (95% CI)
**Parent gender**	1.05 (0.73–1.52)	**-**
**Parent age**	0.98 (0.96–0.99)*	0.98 (0.97–1.01)
**Parent ethnicity (Non-Hispanic White = reference category)**		
Non-Hispanic Black	2.08 (1.23–3.53)*	1.46 (0.79–2.72)
Hispanic	2.61 (1.54–4.42)*	2.16 (1.16–4.01)*
Asian	0.98 (0.36–2.64)	0.71 (0.22–2.31)
Other	1.41 (0.51–3.94)	1.47 (0.44–4.90)
**Religious service attendance**	0.99 (0.87–1.13)	-
**Political views**	1.28 (0.99–1.66)†	1.07 (0.80–1.44)
**Son’s age**	1.01 (0.92–1.10)	-
**Son visited health care provider in past year**	2.22 (1.12–4.40)*	2.46 (1.00–6.06)†
**Number of flu shots son received in past 2 years**	1.82 (1.45–2.26)*	1.72 (1.35–2.19)*
**Son’s health insurance**	0.55 (0.38–0.79)*	0.56 (0.37–0.83)*
**Frequency of discussion about sexual health**	1.61 (1.37–1.89)*	1.64 (1.37–1.97)*

Note: OR, odds ratio; CI, confidence interval.

*p<.05.

† *p<*.10.

## Discussion

The adolescent male vaccination rate reported by this national sample of parents in 2012 was comparable to the 2012 NIS-TEEN data for males and females ages 13 to 17. [4] Although this set of findings indicates a marked increase from the initiation of male vaccination in 2011, it nonetheless points to ongoing under-immunization of the vaccine in males. Hispanic ethnicity of the parent, as well as health care provider visit in the prior year by the son, number of flu shots received by the son, son’s health insurance status, and frequency of discussion about sexual health were all associated with HPV vaccine initiation.

Parent and adolescent demographics have been shown in previous research to predict uptake of the HPV vaccine. Both conservative political views [8] and increased religious service attendance (religiosity) [8], [9] were shown to predict decreased vaccine uptake in females. In this study, a correlation was found between increasing parent age and both conservative political views and religiosity; however, none of these were associated with vaccine uptake. Son’s age was not a predictor of vaccination uptake, a finding consistent with the 2012 NIS-TEEN data on males. [4] In contrast, 2012 NIS-TEEN findings indicate a steady increase in female HPV vaccination rates from 13 through 17 years of age. [4] These findings may indicate that parents are more comfortable with vaccinating young adolescent sons than young adolescent daughters, perhaps reflecting, a sexual double standard, which is a well-established societal phenomenon. [21] Parents may be more willing to vaccinate young males if the idea of emerging male sexuality is less discomforting than emerging female sexuality. It is also possible that HCPs are influenced by the same double standard, as research suggests that frequency of HCP recommendation for HPV vaccine for females also increases with patient age. [22] As rates of male vaccination increase, it will be important to assess whether these differences persist, as they have implications for gender-based health disparities. Past research has suggested that sexual double standards have had a negative effect on women’s sexual health [21].

Consistent with our results, previous studies have found that recent influenza vaccination is related to both acceptability and initiation of HPV vaccination. [8], [16] Both influenza and HPV vaccination rates are relatively low compared to other childhood/adolescent vaccines [4], [23] suggesting that both may suffer from vaccine hesitancy on the part of parents and HCPs.

Both influenza and HPV are non-required vaccines, and parents often decline them for a multitude of reasons. [18], [24], [25] Published research suggests that HCP recommendation and strength of recommendation are associated with increased HPV vaccine uptake. [12], [17], [19] HCPs who strongly recommend all vaccines, not just those required for school, may be increasing HPV vaccination rates within their practice. Future investigation examining the basis for the relationship between influenza vaccine and HPV vaccination may help to clarify the nature of this association, which in turn may have implications for clinical practice. We also found that those sons who have private health insurance are less likely than those without private health insurance to have initiated vaccination. Previous publications representing data on both males and females suggest that having public insurance is related to increased HPV vaccine initiation which may be related to public vaccine programs such as the Vaccines for Children Program [26], [27].

Lastly, it has been proposed that parents who are comfortable discussing sexual health topics with their sons are more likely to engage in conversation with them regarding HPV vaccination. [28] Since parents are, in the end, the consenting party for those sons under age 18, this comfort level with sexual health discussion may be a vital key to improving vaccination rates. [7], [29] Sons may have knowledge, or specific beliefs, about HPV vaccination. By encouraging parents and teens to have open discussions surrounding sexual health, HCPs may provide a forum for discussion surrounding prevention of sexually transmitted infections, and son’s may feel more comfortable expressing their beliefs about HPV vaccination.

As in all research, there were limitations to this study. The survey participants represented a national sample of parents of sons; however, the sample was not nationally representative, and vaccination status was based on parental report which has variable accuracy. [30] These issues can raise concerns regarding generalizability. However, as noted above, the most recent NIS-TEEN data on initiation of HPV vaccine in males is similar to the data we obtained, suggesting that our sample did not deviate substantially from the nationally representative NIS-TEEN sample with respect to vaccination rates [4].

This research shows sons who were exposed to healthcare services, specifically those who had received flu shots, had an increased rate of vaccine initiation. Both the Society for Adolescent Health and Medicine [31], and the American Academy of Pediatrics Bright Futures initiative [32] support routine provision of all recommended vaccines for adolescents, including influenza vaccination. Our findings suggest that HPV vaccination rates might increase by following the practice of routinely recommending all adolescent vaccines. We would encourage further research to examine the relationship between HCP recommendation and receipt of these routine but non-required vaccinations. The possibility of a sexual double standard is intriguing and deserves further investigation.

## Supporting Information

S1 Dataset **Parents of Sons Data Set.**
(SAV)

S1 Manuscript **Acceptability of the human papillomavirus vaccine and reasons for non-vaccination among parents of adolescent sons.** Kelly L. Donahue, PhD, Nathan W. Stupiansky, PhD, Andreia B. Alexander, MD, PhD, MPH, Gregory D. Zimet.
(PDF)
