# Supplementary material for: Vaccinating Sons against HPV: Results from a U.S. National Survey of Parents
Source: PLoS One. 2014 Dec 26;9(12):e115154. doi: 10.1371/journal.pone.0115154 (PMC4277268; doi:10.1371/journal.pone.0115154)
Supplement: S1 Manuscript — Acceptability of the human papillomavirus vaccine and reasons for non-vaccination among parents of adolescent sons. Kelly L. Donahue, PhD, Nathan W. Stupiansky, PhD, Andreia B. Alexander, MD, PhD, MPH, Gregory D. Zimet. (PDF) [file pone.0115154.s002.pdf]

Vaccine 32 (2014) 3883–3885

Contents lists available at ScienceDirect

Vaccine

journal homepage: www.elsevier.com/locate/vaccine

Brief report

Acceptability of the human papillomavirus vaccine and reasons for non-vaccination among parents of adolescent sons

Kelly L. Donahue*, Nathan W. Stupiansky, Andreia B. Alexander, Gregory D. Zimet

Section of Adolescent Medicine, Department of Pediatrics, Indiana University School of Medicine, 410 West 10th Street, Suite 1001, Indianapolis, IN 46202, USA

ARTICLE INFO

Article history:
Received 17 February 2014
Received in revised form 6 May 2014
Accepted 7 May 2014
Available online 18 May 2014

Keywords:
Human papillomavirus
Vaccination
Adolescence
Healthcare providers
Vaccine acceptability

ABSTRACT

Routine administration of the quadrivalent human papillomavirus (HPV) vaccine has been recommended for 11–12-year-old males since 2011, but coverage remains low. In a U.S. national sample of parents of 11–17-year-old males (*n* = 779), 78.6% of parents reported their sons had not received the HPV vaccine. The most common reason for non-vaccination (56.7%) was "My doctor or healthcare provider has not recommended it." Parents citing only logistical reasons for non-vaccination (e.g., lack of recommendation, access, or education, *n* = 384) reported significantly higher vaccine acceptability than parents reporting a combination of attitudinal (e.g., concerns about vaccine safety or efficacy) and logistical barriers (*n* = 92), while parents citing only attitudinal barriers (*n* = 73) reported the lowest level of vaccine acceptability. In sum, many parents are willing but have not vaccinated sons due to logistical barriers, most commonly lack of healthcare provider recommendation. These findings have important implications for increasing HPV vaccination coverage among adolescent males.

© 2014 Elsevier Ltd. All rights reserved.

1. Introduction

Human papillomavirus (HPV) is a highly prevalent sexually transmitted infection [1–3], and HPV vaccination provides effective protection against adverse health outcomes associated with HPV, including anogenital cancers and genital warts [4–6].

Routine HPV vaccination has been recommended for 11–12-year-old females and males since 2006 [7] and 2011 [8], respectively. However, HPV vaccine coverage remains significantly lower than coverage for other routine early-adolescent immunizations, with males less likely than females to initiate and/or complete the three-dose HPV vaccine series [9]. In 2012, 20.8% of 13–17-year old males in the U.S. received one or more doses of the HPV vaccine, compared to 53.8% of 13–17-year-old females [9].

Recommendation from a healthcare provider is a key predictor of HPV vaccine initiation [10–12]. Parents' and healthcare providers lack of knowledge or misperceptions regarding HPV infection and the need for vaccination among males, in conjunction with the relative recency of routine HPV vaccination for adolescent males, may contribute to this gender gap in vaccine coverage [10,11].

Previous research regarding HPV vaccination of adolescent females has identified additional logistical obstacles (e.g., vaccine cost or healthcare access) and negative attitudes about vaccination (e.g., concerns about safety or side effects) [10,13,14] as barriers affecting immunization coverage. Previous research also suggests that young-adult females reporting attitudinal barriers to HPV vaccination report lower intent to vaccinate relative to women reporting logistical barriers [14].

The objective of this study was to explore reasons for non-vaccination and vaccine acceptability (i.e., willingness to vaccinate) among parents whose adolescent sons had not received the HPV vaccine. This study also examined whether the type of barriers to vaccination endorsed by parents (i.e., logistical and/or attitudinal barriers) was associated with willingness to vaccinate.

2. Methods

2.1. Sample

Data were collected in July–August 2012 through a Web-based survey assessing HPV-vaccination-related attitudes, beliefs, and behaviors in a national sample of parents of 11–17-year-old males in the U.S. The study was approved by the IRB at Indiana University – Purdue University Indianapolis. Data collection was facilitated by Survey Sampling International (SSI), a survey research company

* Corresponding author. Tel.: +1 317 274 2905.
E-mail address: kldonahu@iu.edu (K.L. Donahue).

http://dx.doi.org/10.1016/j.vaccine.2014.05.035
0264-410X/© 2014 Elsevier Ltd. All rights reserved.

Table 1
Parents' reported reasons for non-vaccination of 11–17-year-old sons and associated HPV vaccine acceptability.

Reason for non-vaccination		# of parents endorsing	% of parents reporting 0 doses received[a]	Acceptability of HPV vaccination among parents endorsing reason	
				M	SD
1	I don't believe the shot works.[b]	32	5.8%	17.3	24.09
2	I think the HPV vaccine could be dangerous for my son.[b]	83	15.0%	26.7	24.80
3	I believe the shot will have side effects.[b]	98	17.7%	32.4	29.96
4	I believe the shot will make it easier for my son to have sex.[b]	47	8.5%	48.4	31.02
5	My son decided he did not want the vaccine.[c]	33	5.9%	51.5	30.56
6	I don't have insurance or money to pay for the shot.[c]	40	7.2%	52.7	26.65
7	My doctor or healthcare provider has not recommended it.[c]	338	60.9%	57.4	26.35
8	I didn't know the vaccine was for males.[c]	180	32.4%	59.2	27.00
9	We have not seen a doctor or healthcare provider in a long time.[c]	56	9.4%	60.3	26.90

HPV: human papillomavirus; M: mean; SD: standard deviation.

[a] Total possible $n = 555$; Parents indicating that they did not know how many doses of the vaccine their son had received ($n = 40$) were not asked questions regarding barriers to vaccination. Mean acceptability among these parents was 69.4 with SD = 24.5. An additional 6 parents who reported that their son had received 0 doses did not select any of the provided reasons for non-vaccination but are included here in the denominator.

[b] Categorized as attitudinal barriers to vaccination in analyses.

[c] Categorized as logistical barriers to vaccination in analyses.

that maintains national panels of adults in 37 countries. Each panel member may participate in up to four surveys annually, and participants are entered into a lottery to win a monetary prize through SSI. E-mail invitations were sent at random to members of SSI's U.S. panel meeting the study's target demographic (i.e., parents with at least one 11–17-year-old son in the household). Initially, 1322 panelists responded to the e-mail invitation to participate in the current survey, with 803 panelists meeting the eligibility requirement, and 779 of eligible panelists (97%) completing the survey. Respondents with more than one 11–17-year-old son were prompted to answer items about their youngest son in the age range. While participants for the study were recruited nationally, they do not constitute a nationally representative sample.

2.2. Measures

Son's HPV vaccination history was assessed using parent report of the number of HPV shots their son had received. Participants missing vaccination history data ($n = 21$) were excluded from analyses. Parents who reported their son had zero doses or who were not sure if their son had received any doses of the HPV vaccine were categorized as "non-vaccinators."

Acceptability of HPV vaccination was assessed by asking non-vaccinators to indicate their willingness to vaccinate their son if the HPV vaccine were available in a clinic or their doctor's office (i.e., vaccine acceptability) using a scale of 0 (absolutely not willing) to 100 (absolutely willing). Parents selected from multiples of 10 within this range. Parents were asked to report willingness to vaccinate at three different cost levels: free, $30/dose, and $120/dose, reflecting costs in the context of public funding, insurance co-pay, and out-of-pocket costs, respectively. Within-parent reliability across cost levels was high (Chronbach's $\alpha = .86$), with parents' mean vaccine acceptability across the three cost levels used in the presented analyses.

Parents reporting zero doses then selected from among nine reasons describing why their son had not received the HPV vaccine (see Table 1), based on common barriers to vaccination that have been identified previously [12,15,16]. Parents were instructed to select all applicable reasons.

2.3. Statistical analysis

The frequency of each reported reason for non-vaccination was calculated as well as mean vaccine acceptability among parents endorsing each reason. Parents were then categorized into three groups reflecting reasons for non-vaccination: parents who endorsed only one or more attitudinal barrier(s), parents who endorsed only one or more logistical barrier(s), and parents who endorsed a combination of attitudinal and logistical barriers. Group differences in vaccine acceptability were then explored using univariate ANOVA.

3. Results and discussion

Of parents completing the survey, 51.5% were female and 48.5% were male, with mean (*M*) age 42.2 years (range 18–70). Parents described their own race/ethnicity as white (73.8%), African American/Black (10.0%), Hispanic/Latino (9.8%), Asian (3.6%), or other (2.8%); and 75.5% of parents had completed at least some years of college education.

Non-vaccinators comprised the majority of the sample (78.6%), with $n = 555$ reporting their son had received zero doses and $n = 40$ reporting they were not sure if their son had received any doses. Of parents reporting vaccine initiation (21.4%), receipt of 1, 2, or 3 doses was reported by $n = 89$, $n = 49$, and $n = 24$ parents, respectively. Among non-vaccinators, mean vaccine acceptability was 55.2 (SD = 29.7, range 0–100).

Table 1 indicates the number of parents endorsing each reason for non-vaccination, mean vaccine acceptability among parents endorsing that reason, and the corresponding barrier-type category. Only parents reporting their son had received zero doses of the vaccine were asked to select reasons for non-vaccination. Overall, non-vaccination against HPV was most often the result of logistical barriers – in particular, the most common reason for non-vaccination (endorsed by 60.9%) was "My doctor or healthcare provider has not recommended it." The majority of parents (57.7%) cited one reason for non-vaccination; 24.7%, two reasons, and 16.4%, three or more. Six parents (1.1%) selected no reasons for non-vaccination and were excluded from subsequent analyses.

Non-vaccinators with available data regarding barriers to vaccination ($n = 549$) were then divided into three groups, reflecting parents endorsing only logistical barriers to vaccination ($n = 384$, 69.9%), only attitudinal barriers ($n = 73$, 13.2%), and a combination of logistical and attitudinal barriers ($n = 92$, 16.7%). There were no differences between the three groups with regard to parent age, gender, race/ethnicity, or education. Vaccine acceptability differed significantly across the three groups, $F(2,546) = 62.87$, $p < .001$. Post hoc comparisons between groups were conducted using the Games–Howell test to account for unequal sample sizes and variances across groups. Parents endorsing only logistical barriers

($M = 62.1$, 95% confidence interval (CI) [59.4, 64.8]) reported significantly higher vaccine acceptability than the combination group ($M = 42.5$, 95% CI [37.0, 48.0]), $p < .001$, as well as the attitudinal-only group ($M = 26.7$, 95% CI [20.5, 32.9]), $p < .001$. The combination group also reported significantly higher vaccine acceptability than the attitudinal-only group ($p = .003$).

These results suggest that parents reporting logistical obstacles to HPV vaccination, even in the context of some attitudinal concerns, represent an important opportunity for increasing HPV vaccine coverage in the United States, considering the relatively high prevalence of these parents and their higher level of vaccine acceptability. Interventions aimed at reducing these logistical barriers to vaccination, such increasing the strength and/or occurrence of provider recommendation and increasing access to preventative care during adolescence [17] – may help eliminate a significant proportion of missed immunization opportunities for adolescent males. Once such logistical barriers are addressed, providers who are prepared with information to appropriately address concerns about vaccine safety or efficacy during interactions with the minority of parents endorsing such concerns may be successful in achieving parental agreement to vaccinate [18,19].

There are several limitations to this study. First, data were not collected from a nationally representative sample, and generalizability of these results to the broader population of American parents of adolescent sons may be limited. Because data are unavailable regarding the initial number of panel members receiving the e-mail invitation for participation, the true response rate is unknown. Additionally, analyses relied on parent recall of son's vaccination history, and parents may be prone to underestimate vaccine coverage [20]. However, despite these limitations, the rate of vaccine initiation obtained in the current study (21.4%) is very similar to rates obtained from medical records from a nationally representative sample of adolescent males (20.8%), [9] with data for both studies collected at similar time periods. Additionally, findings regarding parental willingness to vaccinate may represent conservative estimates of acceptability due the inclusion of the $120/dose cost scenario, which was associated with lower vaccine acceptability. This direct-cost amount should be encountered infrequently by U.S. parents due to insurance coverage of routine immunizations and federally funded vaccine programs for uninsured or underinsured children.

4. Conclusions

Many parents report being willing to vaccinate their adolescent sons against HPV but have not yet vaccinated their sons due to logistical barriers – most commonly, lack of recommendation from a healthcare provider. Minimizing barriers to vaccination, such as increasing adolescent access to low- or no-cost immunization and improving provider communication with parents about the importance of vaccinating young males against HPV, may help to increase vaccination coverage in this population.

Acknowledgments

This study was supported by Merck & Co., Inc. through a grant (IISP #38094) to Gregory D. Zimet. Manuscript contents are the sole responsibility of the authors and do not reflect the official view of Merck & Co., Inc. Merck & Co., Inc. did not have a role in study design; analysis and interpretation of data; writing the report; or the decision to submit the report for publication. Contributions to survey conception and design by Andreia Alexander were supported by R25CA117865-05S1 from the National Cancer Institute and the Continuing Umbrella of Research Experience (CURE) Program. Analyses were conducted by Kelly L. Donahue, Ph.D., through postdoctoral support provided to the Center for HPV Research by the Indiana University – Purdue University Indianapolis Signature Centers Initiative. Preliminary versions of the analyses in this manuscript will be presented by Dr. Donahue at the University of Michigan Health System & Adolescent Health Initiative Conference on Adolescent Health in April 2014.

Conflict of interest: Gregory Zimet has been an investigator on investigator-initiated research funded by Merck, Inc. and has received an unrestricted program development grant from GlaxoSmithKline. Nathan Stupiansky has been an investigator on investigator-initiated research funded by Merck, Inc. Kelly Donahue and Andreia Alexander have no financial disclosures to report.

References

[1] Giuliano AR, Lu B, Nielson CM, Flores R, Papenfuss MR, Lee JH, et al. Age-specific prevalence, incidence, and duration of human papillomavirus infections in a cohort of 290 US men. J Infect Dis 2008;198:827–35.
[2] Dunne EF, Unger ER, Sternberg M, McQuillan G, Swan DC, Patel SS, et al. Prevalence of HPV infection among females in the United States. JAMA 2007;297(8):813–9.
[3] Forhan SE, Gottlieb SL, Sternberg MR, Xu F, Datta SD, McQuillan GM, et al. Prevalence of sexually transmitted infections among female adolescents aged 14–19 in the United States. Pediatrics 2009;124(6):1505–12.
[4] Baandrup L, Blomberg M, Dehlendorff C, Sand C, Andersen KK, Kjaer SK. Significant decrease in the incidence of genital warts in young Danish women after implementation of a national human papillomavirus vaccination program. Sex Transm Dis 2013;40(2):130–5.
[5] Giuliano AR, Palefsky JM, Goldstone S, Moreira Jr ED, Penny ME, Aranda C, et al. Efficacy of quadrivalent HPV vaccine against HPV infection and disease in males. N Engl J Med 2011;364(5):401–11.
[6] Hariri S, Markowitz LE, Dunne EF, Unger ER. Population impact of HPV vaccines: summary of early evidence. J Adolesc Health 2013;53(6):679–82.
[7] Centers for Disease Control and Prevention. Quadrivalent human papillomavirus vaccine: Recommendations of the ACIP. MMWR Morb Mortal Wkly Rep 2007;56 (Early Release):1–24.
[8] Centers for Disease Control and Prevention. Recommendations on the use of quadrivalent human papillomavirus vaccine in males – Advisory Committee on Immunization Practices (ACIP). MMWR Morb Mortal Wkly Rep 2011;60(50):1705–8.
[9] Centers for Disease Control and Prevention. National and state vaccination coverage among adolescents aged 13–17 years – United States, 2012. MMWR Morb Mortal Wkly Rep 2013;62(34):685–93.
[10] Zimet GD, Rosberger Z, Fisher WA, Perez S, Stupiansky NW. Beliefs, behaviors and HPV vaccine: correcting the myths and the misinformation. Prev Med 2013;57:414–8.
[11] Reiter PL, McRee AL, Pepper JK, Gilkey MB, Galbraith KV, Brewer NT. Longitudinal predictors of human papillomavirus vaccination among a national sample of adolescent males. Am J Public Health 2013;103:1419–27.
[12] Holman DM, Benard V, Roland KB, Watson M, Liddon N, Stokley S. Barriers to human papillomavirus vaccination among US adolescents: a systematic review of the literature. JAMA Pediatrics 2013:E1–7.
[13] Darden PM, Thompson DM, Roberts JR, Hale JJ, Pope C, Naifeh M, et al. Reasons for not vaccinating adolescents: National Immunization Survey of Teens, 2008–2010. Pediatrics 2013;131(4):645–51.
[14] Gerend MA, Shepherd MA, Shepherd JE. The multidimensional nature of perceived barriers: global versus practical barriers to HPV vaccination. Health Psychol 2013;32(4):p361.
[15] Stupiansky NW, Alexander AB, Zimet GD. Human papillomavirus vaccine and men: what are the obstacles and challenges? Current Opin Infect Dis 2012;25(1):86–91.
[16] Kester LM, Zimet GD, Fortenberry JD, Kahn JA, Shew ML. A national study of HPV vaccination of adolescent girls: rates, predictors, and reasons for non-vaccination. Matern Child Health J 2013;17(5):879–85.
[17] Wong CA, Taylor JA, Wright JA, Opel DJ, Katzenellenbogen RA. Missed opportunities for adolescent vaccination, 2006–2011. J Adolesc Health 2013;53(4):492–7.
[18] Kempe A, Daley MF, McCauley MM, Crane LA, Suh CA, Kennedy AM, et al. Prevalence of parental concerns about childhood vaccines: the experience of primary care physicians. Am J Prev Med 2011;40(5):548–55.
[19] Opel DJ, Heritage J, Taylor JA, Mangione-Smith R, Salas HS, DeVere V, et al. The architecture of provider–parent vaccine discussions at health supervision visits. Pediatrics 2013;132(6):1037–46.
[20] Stupiansky NW, Zimet GD, Cummings T, Fortenberry JD, Shew M. Accuracy of self-reported human papillomavirus vaccine receipt among adolescent girls and their mothers. J Adolesc Health 2012;50(1):103–5.
